# Factors influencing motivation and job satisfaction among supervisors of community health workers in marginalized communities in South Africa

**DOI:** 10.1186/s12960-016-0151-6

**Published:** 2016-09-06

**Authors:** Olagoke Akintola, Gamuchirai Chikoko

**Affiliations:** 1 School of Applied Human Sciences, University of KwaZulu-Natal, Mazisi Kunene Road, Glenwood, Durban 4041 South Africa; 2 School of Human and Social Development, Nipissing University, 100 College Drive, ON P1B 8L7 North Bay, Canada

**Keywords:** Attrition, Community health worker, Community-based organization, Job satisfaction, Motivation, Primary health care, Retention, Supervision, South Africa

## Abstract

**Background:**

Management and supervision of community health workers are factors that are critical to the success of community health worker programmes. Yet few studies have explored the perspectives of supervisors in these programmes. This study explored factors influencing motivations of supervisors in community health worker programmes.

**Methods:**

We conducted qualitative interviews with 26 programme staff providing supervision to community health workers in eight community-based organizations in marginalized communities in the greater Durban area of South Africa from July 2010 to September 2011.

**Results:**

Findings show that all the supervisors had previous experience working in the health or social services sectors and most started out as unpaid community health workers. Most of the participants were poor women from marginalized communities. Supervisors’ activities include the management and supply of material resources, mentoring and training of community health workers, record keeping and report writing. Supervisors were motivated by intrinsic factors like making a difference and community appreciation and non-monetary incentives such as promotion to supervisory positions; acquisition of management skills; participation in capacity building and the development of programmes; and support for educational advancement like salary, bonuses and medical benefits. Hygiene factors that serve to prevent dissatisfaction are salaries and financial, medical and educational benefits attached to the supervisory position. Demotivating factors identified are patients’ non-adherence to health advice and alienation from decision-making. Dissatisfiers include working in crime-prevalent communities, remuneration for community health workers (CHWs), problems with material and logistical resources, job insecurity, work-related stressors and navigating the interface between CHWs and management. While participants were dissatisfied with their low remuneration, they were not demotivated but continued to be motivated by intrinsic factors. Our findings suggest that CHWs’ quest for remuneration and a career path continues even after they assume supervisory positions. Supervisors continue to be motivated to work in mid-level positions within the health and social services sectors.

**Conclusions:**

Global efforts to develop and increase the sustainability of CHW programmes will benefit immensely from insights gained from an exploration of supervisors’ perspectives. Further, national CHW programmes should be conceptualized with the dual purpose of building the capacity of CHWs to strengthen health systems and reducing unemployment especially in marginalized communities with high unemployment and low-skilled labour force.

## Background

Primary health care (PHC) is a philosophy and key strategy for achieving universal access to health care [1]. In many low- and middle-income countries (LMICs), the adoption of the PHC approach has been characterized by the use of community health workers (CHWs). CHWs typically receive basic health training and provide health and social services in the communities where they live [2]. Globally, there has been a renewed interest in the use of CHW due to health worker shortages, the burden of HIV and AIDS on the health systems and the need to achieve the millennium development goals particularly in LMICs [2, 3]. CHW programmes have been shown to be effective in improving health outcomes particularly among marginalized populations in LMICs [4, 5].

In South Africa, most CHWs are recruited and trained by community-based organizations (CBOs), many of whom are funded by government agencies [2, 6–8]. They help in preventative activities and in the delivery of health services such as maternal and child health services, home-based care for people living with AIDS and chronic illnesses, antiretroviral therapy and TB treatment [2, 3]. CHWs also help in addressing the social determinants of health such as poverty, housing, food and education [8, 9].

CHWs working in CBOs in South Africa are managed and supervised by programme staff that include project managers, coordinators, facilitators and nurses [10]. Unlike CHWs who sometimes receive stipends or work without remuneration, supervisors working in CBOs are usually paid salaries [10]. Currently, the South African Department of Health (DOH) is piloting a reform of the PHC system. A key element of this reform is the incorporation of CHWs into the formal health care system as part of ward-based outreach teams that will facilitate access to health care services in households in marginalized communities across the country [6, 11]. Given the central role that CBOs play in the delivery of health care services to marginalized communities, one would expect CHWs and supervisors working in CBOs to play a critical role in the new PHC initiative. However, the new PHC re-engineering policy does not make explicit what their roles will be in the new PHC initiative [9, 12].

Globally, there is a growing body of work on motivations among CHWs. Motivation is defined as ‘an individual’s degree of willingness to exert and maintain an effort towards an organization’s goals’ [13]. An important factor influencing motivation among CHWs and the sustainability of CHW programmes, as demonstrated by several studies, is the management and supervision of CHWs [14–16]. Research has found that the main factors motivating CHWs are altruism, religious and moral obligations, the prospects of paid employment in the health and social services sector, previous experience with HIV and AIDS in the family, and the wish to avoid idleness [10, 11, 17]. Studies have also identified supervision as a key factor influencing retention among CHWs [18, 19]. CHWs participating in a recent study in Mozambique indicated that they felt demotivated by the supervision provided because it was infrequent and irregular and focussed on fault finding [16].

In CBOs, supervisors work at the interface between the management of CBOs and CHWs [10, 16]. This position could enable them to gain deep insights into issues relating to CHWs, supervision and the management of CHW programmes, which could be critical to the success or failure of these programmes. One could argue, therefore, that supervisors’ perspectives should be important considerations in our quest to better understand and scale-up CHW programmes. Yet, there are few studies globally that have sought to understand CHW programmes specifically from the perspective of supervisors [14, 20, 21]. One recent review of literature published in 2015 found that only one article was published on supervision post-2010 [21]. We therefore know very little about the perspectives of supervisors: their characteristics, what their work entails and the factors that influence motivation and job satisfaction among them.

Most of the few available studies focus on supervision within the context of national primary health care programmes [14, 20] while neglecting supervision in CBOs. Yet CBOs manage the majority of CHW programmes globally [2, 8]. If CHW programmes are to provide adequate management and supervision of CHWs, it is critical that they are able to attract, satisfy and retain their supervisors. It is therefore imperative to understand the perspectives of supervisors in CHW programmes.

An understanding of factors influencing motivation among supervisors could help inform health policy and organizational interventions aimed at improving satisfaction and retention of supervisors as well as the effectiveness and sustainability of CHW programmes. Yet little is known about job satisfaction and motivation among supervisors working in CHW programmes. In this study, we sought to understand the factors influencing motivation and job satisfaction among supervisors of CHWs affiliated to CBOs in South Africa.

This study draws on Herzberg’s dual-factor theory of motivations, also known as the motivation-hygiene theory [22], to help illuminate factors influencing motivation and job satisfaction among supervisors in CHW programmes in South Africa. The motivation-hygiene theory postulates that job satisfaction and dissatisfaction are not opposite ends of the same continuum but instead represent two distinct and separate continua representing two dimensions of job motivation. According to the theory, factors that motivate individuals at work are intrinsic to the work itself and rewards that flow from the performance of that work. These are factors associated with individuals’ need for self- actualization and self-realization in work and include achievement, responsibility, growth or advancement, recognition, autonomy, promotion and the work itself, referred to as *motivators* or sometimes as *satisfiers*. The presence of these factors leads to job motivation, and their absence leads to demotivation but not to dissatisfaction. Herzberg and colleagues argue that the factors that lead to job dissatisfaction are extrinsic and relate not to the job itself but to the environment or context of the job. These factors are called *hygiene factors* or *dissatisfiers. Hygiene factors* include working conditions, salary, job security, company policy and administration, supervision and interpersonal relationships [22]. The presence of *hygiene factors* can prevent poor performance but cannot improve productivity. Only the presence of motivation factors (*motivators* or *satisfiers*) can improve productivity. It follows therefore from the motivation-hygiene theory that, in order to motivate an individual, the job itself must be challenging and be of interest to the individual doing the job.

By drawing on Herzberg’s theory, the study aims to illuminate factors that motivate supervisors, thereby helping to gain insights into some of the factors influencing the sustainability of CHW programmes through the lens of supervisors. Although this theory has been critiqued for oversimplifying the relationship between motivation and satisfaction and sources of motivation and dissatisfaction [23], it remains one of the most influential theories for explaining motivation and job satisfaction globally [24] and has been applied extensively in many fields including among CHWs [25–27].

## Methods

### Study setting and context

This study was conducted among supervisors working in CBOs in marginalized communities in the Durban metropolis. The CBOs help fill important gaps in government services delivery by providing health care services as well as social and developmental services to the communities that have limited access to government services. These are poor communities with high unemployment rates and low skills base, where most people are dependent on government grants for income.

All CBOs provide home-based care for people living with HIV/AIDS/TB and other chronic diseases. They also provide health promotion and education, HIV counselling and testing, TB treatment adherence support, care and support for orphan and vulnerable children, day care and drop-in services, psychosocial support, feeding programmes, youth and community development initiatives, paralegal services especially for survivors of rape and support in accessing government health and social services.

### Study design and participants

A qualitative research design was used in this study because of its ability to generate in-depth information about the experiences of supervisors of CHWs [28]. CBO staff providing supervision to CHWs constituted the study population because we believed that they were the most suitable to provide information about factors influencing their motivations. CBOs were selected purposively to reflect a range of CBOs working on health and social services in communities in the Durban metropolis. We had hoped to recruit equal numbers of CBOs from peri-urban and rural communities in the study area. However, we were unable to achieve this balance because there are very few rural areas in the Durban metropolis. Our sample therefore comprised one organization working in an informal settlement; the seven remaining CBOs were drawn from peri-urban communities (Table 1).

We used snowball sampling to select CBOs by first contacting two organizations that we had worked with previously who in turn referred us to other organizations in their network. The managers of the CBOs informed all the supervisors about the study and worked with the research team to arrange interview dates. We then used purposive sampling to recruit participants if they were employed in a CBO and provided direct supervision to CHWs for at least 1 year prior to the study. All of the supervisors invited to participate in the study agreed. However, four supervisors—two each from two CBOs—could not participate because they were not available during the time set for the interviews due to work commitments. A total of 26 interviews were conducted with supervisors who were available on the pre-arranged interview dates. Table 2 shows the socio-demographic characteristics of the participants.

**Table 1:** Participants interviewed in community-based organizations

Community-based organization	Location	Type of area	No of participants interviewed
A	Marianhill	Peri-urban	6
B	Amanzimtoti	Peri-urban	5
C	Nazareth	Peri-urban	4
D	Clermont	Peri-urban	3
E	Umlazi	Peri-urban	2
F	Siyanda	Informal settlement	2
G	Lamontville	Peri-urban	2
H	Kwamashu	Peri-urban	2

**Table 2:** Profile of supervisors

Variable	Frequency	Percent
Gender		
Male	3	11.5
Female	23	88.5
Age (years)		
25–29	2	7.7
30–34	3	11.5
35–39	5	19.2
40–44	7	26.9
45–49	5	19.2
50–54	2	7.7
55–59	1	3.8
60–64	1	3.8
Experience in CBO (years)		
2–4	5	19.2
5–9	11	42.3
10–11	8	30.8
13–15	2	7.7
Designation		
Home-based care coordinator	5	19.2
Orphan and vulnerable children coordinator	3	11.5
Area coordinator	5	19.2
Community facilitator	3	11.5
CBO manager	7	26.9
Nurse supervisor	2	7.7
Agricultural supervisor	1	7.7

### Data collection

Qualitative data for the study was collected with semi-structured interviews [23]. We used semi-structured interview schedules, which allowed interviewers’ flexibility, to collect in-depth information. The interview schedule offered interviewers the opportunity to ask probing questions. The interview schedule was developed after an extensive review of the literature on CBOs, CHWs and supervision in primary health care as well as Herzberg’s theory on motivation. Thereafter, we extracted particular themes from the literature which relates to motivation of supervisors and used them to frame open-ended questions. These questions covered the following themes: demographic information of participants, job description and factors influencing motivation.

With respect to factors influencing motivation, we used themes extracted from our review of Herzberg’s theory and the literature on CHWs and CBOs in South Africa to generate a list of potential *motivators* and *satisfiers* in community-based care, which served as prompts to guide our interview. For example, we asked about participants’ experiences regarding the role of the different aspects of the job such as the job itself, achievement in their supervisor position, recognition, career advancement and responsibility within the organization, in motivating (or demotivating) them and then repeated the question in relation to a list of hygiene factors that are in the environment of the job such as supervision, interpersonal relations, organizational policy, working conditions, security and status. The interviews were conducted in the participants’ offices in either isiZulu or English by four interviewers (one of the authors and three other trained interviewers) who were fluent in isiZulu. Each participant was interviewed by two interviewers. The interviews were conducted from July 2010 to September 2011 and took between 35 and 65 min.

Ethical clearance was obtained from the Human Sciences Research Ethics Committee of the University of KwaZulu-Natal. In addition, all the CBOs approved of the study. Thereafter, we sought and obtained written informed consent from each participant after the interviewers provided detailed explanation about the study to each of them, assuring confidentiality and anonymity of information collected. The interviewers also obtained permission from the participants to conduct audio-recording of the interviews.

### Data analysis

The data was transcribed verbatim, and the data in isiZulu was translated into English by two of the people who conducted the interviews and are native isiZulu speakers. We used the principles of thematic analysis to analyse the data. Two assessors (one of whom is an author: GC) conducted the analysis separately following the five steps recommended by Braun and Clarke [28]. This entailed first familiarizing themselves with the data after which they proceeded to develop codes. Next, each of the assessors developed themes from the various codes and then reviewed and named the themes [28, 29]. Once these steps were completed, the assessors compared the results of the analyses and made revisions using an iterative process. Both assessors reached consensus before each theme was named.

We conducted member checks in order to improve the quality of the data and the rigour of the analysis [30, 31]. Creswell recommended the presentation of ‘polished findings’ in form of themes or patterns to research participants in order to receive feedback and improve rigour when conducting member checks [30]. Following Creswell (2009), we presented the emerging themes from our analysis to two supervisors in each of the CBOs. The supervisors were nominated by the other supervisors to represent them. We scheduled meetings with the supervisor representatives where we presented the preliminary themes from our data analysis and invited comments and clarifications. The feedback from the participants was integrated into the analysis process. In order to further harmonize the analysis and minimize bias, a third assessor (also an author: OA), who did not participate in the initial analysis, reviewed all the themes, and all three assessors reached consensus after extensive discussions and revisions were made to the themes.

## Results

The results are organized into four main themes identified from the analysis of the data: Who are the supervisors? What do supervisors do? What factors influence job satisfaction and motivation among supervisors? and What factors influence job dissatisfaction and demotivation among supervisors?

### Who are the supervisors in CBO-run CHW programmes?

As shown in Table 2, an overwhelming majority of the supervisors were women. The participants’ ages ranged from 25 to 64 years, and their years of experience ranged from 2 to 15 years. Most of the participants worked in small CBOs with limited financial resources and therefore could not hire enough supervisors. They were therefore combining the roles of CBO managers with that of supervisors. All the supervisors had some previous experience working in the health, social or development sector (Table 3); the majority had worked for many years as CHWs in the same CBO before being promoted to a supervisory position. Others had worked in a more professional position as community development workers, nurses and social workers. One participant who worked for many years as a trained social worker with the government resigned after she started her own CBO in response to the high burden of AIDS in her community.

**Table 3:** Work experience of supervisors

Previous work	Frequency	Percentage
Former CHW	17	65.4
Former community development worker	2	7.7
Former nurse	2	7.7
Former social worker	2	7.7
Nurse (current)	1	3.8
Nurse (retired)	2	7.7

I am a social worker by profession and responding to social welfare issues and problems in this country such as AIDS becomes unavoidable because of the high prevalence rate here in South Africa. (Manager/supervisor)

Two of them had worked as nurses in government hospitals but now working as nurse/supervisors for their CBOs. One of them initially worked as a CHW, and the CBOs provided her with financial and moral support to train as a nurse after which she was employed as a nurse with the same CBO.

CHWs are usually promoted to supervisory positions when the positions become available. The criteria used include length of service, track record of performance as CHW and a review of the assessments of their supervisors. The initial recommendation is done by current supervisors who submit the names of top performing CHWs to the CBO management. Thereafter, the CHWs are requested to apply for the position of supervisors and then go through a formal interview process to assess their suitability for the position of supervisor. There are typically many more CHWs who desire to become supervisors and meet the minimum requirements than there are positions. Therefore, only the best performing CHWs get selected after interviews, and unsuccessful candidates are requested to wait for other opportunities.

In small CBOs that did not have funding to support the salaries of supervisors, the managers of the CBOs combined the role of a project manager with that of a supervisor. Five of the managers were the founders of the CBOs and had experience in nursing, social work, community development or community health. One of the founders who trained as a nurse quit her hospital job to avoid clinical practice in a health facility choosing instead to work in a position in community-based care that afforded her less frequent contact with patients.

I actually didn’t want to go and see the patients because it (trauma) stays with me, I can’t stand it but wanted to help towards caring for the patients. That’s why I decided to do community work instead of hospital work. (Manager/supervisor)

### What is the role of supervisors in CBO-run CHW programmes?

Most of the participants have some experience working in community health. However, all the participants receive induction training after they are recruited and subsequently participate in training workshops on various topics related to their work. Most of these workshops are train-the-trainer workshops that enable them to acquire knowledge and skills essential for training, managing and overseeing the work of CHWs, keeping records and writing reports.

Supervisors’ main role is to facilitate and provide oversight for the work of CHWs. Each was responsible for supervising between 10 and 25 CHWs. Supervisors make regular visits (on average 4 days a week) to the communities to provide supplies to CHWs, review their work and provide support for them. They also provide training to new CHWs and on-going training to current CHWs. Table 4 describes the full range of the activities performed by supervisors. New functions are added regularly as the CBOs develop new programmes or interventions to address the needs of the communities that they serve. The supervisors receive regular training to update their knowledge and skills in order to perform the new tasks. In addition, training is also provided to address new challenges that arise in the community that are added to their job description. The most commonly mentioned supervisory activities are medical and material supply and management, training of CHWs, mentoring and support for CHWs, record keeping, report writing and problem solving. Some of the senior supervisors (with more years of experience) help with the development of training modules while those working with larger CBOs who had many years of experience conduct training sessions for CHWs in smaller CBOs for a fee.

**Table 4:** Supervisory activities performed by participants

Activity	Description
Administration and record keeping	Overall responsibility for the management and administration of area covered and keeping vital statistics related to set indicators
Data collection/collation	Collating data on key indicators and measures
Report writing	Writing comprehensive reports of all activities occurring under their area of jurisdiction
Stock management	Managing stock and ensuring adequate supply of materials is in stock
Resources and supplies management	Supplying and monitoring of materials and food for onward distribution
Problem solving/joint problem solving	Solving problems that arise from the day-to-day activities of CHWs, sometimes jointly with CHWs
Mentoring CHWs	Providing mentoring to CHWs and serving as a role model
Appraisal of CHWs	Conducting appraisals and evaluation of CHWS. Recommending CHWs for incentives, awards and promotion
Identifying knowledge and skills gaps	Identifying gaps in knowledge and skills in performance of daily duties through observations/questions and report backs
Formal training	Training CHWs in formal sessions/workshops
On-the-job training	Imparting new skills and knowledge on-the-job
Induction/follow-up training	Showing new CHWs the rope and conducting refresher training on-the-job
Training module development	Developing modules to address identified gaps in knowledge and skills
Providing assistance and support to CHWs	Assisting CHWs with duties and providing support to and filling in for CHWs
Role clarification	Clarifying roles to make sure CHWs stays within job description
Feedback	Receiving feedback about patients and beneficiaries/other programme activities
Decision-making	Making decisions on issues referred by CHWs
Offering advice to management	Reporting to management with options for decision-making
Assessment of health of patients	Assessing patients referred by CHWs
Assessment of socio-economic status of beneficiaries	Assessment of socio-economic status of beneficiaries in order to recommend material and other in-kind support

Although the CBOs stated that they needed nurses to work with CHWs, only the three largest organizations could afford to employ dedicated nurses. The nurses’ main function is to supervise and train CHWs on how to screen and refer patients to the nurses for assessment and treatment and if necessary to make referrals to the health facilities. In the organizations that do not have nurses, the CHWs refer patients to the local clinics while in the three organizations that employ nurses, the CHWs refer patients to their supervisors who work closely with the nurse supervisors on regular rounds to households in the communities. Nonetheless, the supervisors and nurses also make special visits to deal with urgent cases or emergencies when requests are received from CHWs.

I go with home-based carers (CHWs) who have minimum medical education and training…I teach them so that from the patient’s history and the signs and symptoms, they can quickly pick up what is happening and whether and where to refer. (Nurse/supervisor)

Supervisors provide mentoring and support to CHWs who need mentoring as well as assistance and support to deal with challenging situations that they encounter in their daily work. They also help provide advice and practical support in dealing with difficult patients and with general problems.

We go out and I take one of the caregivers with me and tell them that if anyone has a problem with their patient we can go and check because sometimes you find that they have difficult patients. (Manager/supervisor)

In addition, supervisors serve as intermediaries between the CHWs and the management of the CBOs by helping to solve difficult problems confronting CHWs. In cases where supervisors are unable to solve these problems, they help convey these and other grievances from the CHWs to the management. In the same manner, supervisors help convey information, instructions and messages from the management to the CHWs.

### Factors influencing motivation and job satisfaction among supervisors of CHWs

Following Herzberg’s motivation-hygiene theory, we identified two overarching themes: factors facilitating motivation and job satisfaction and factors contributing to job demotivation and dissatisfaction among supervisors (Table 5).

**Table 5:** Factors influencing motivation and job satisfaction among supervisors

Levels of influence	Motivating factors		Hygiene factors	
	Motivators/satisfiers	Demotivators	Non-dissatisfiers (factors preventing dissatisfaction)	Dissatisfiers (factors promoting dissatisfaction)
Community level	• Nature of community work • Making a difference and community appreciation	• Non-adherence to health advice among community members		• Working in crime-prevalent communities
Organizational/programme level	• Promotion to supervisory position • Acquisition of management skills and experience • Participation in capacity building • And the development of programmes • Educational advancement opportunities	Alienation from decision-making	Salaries (supervisory position), retirement benefits, salary advances, financial support, educational loans and medical services	• Remuneration for CHWs • Problems with material and logistical resources • Confronting job insecurity • Dealing with work-related stressors • Navigating the interface between CHWs and management
Policy level (government and donor agencies)				• Shortage of resources and funding for stipends and salaries • Challenges of partnering with government agency

### Factors facilitating motivation and job satisfaction

The factors identified as facilitating motivation of supervisors are discussed under two broad system level themes: community level factors and organizational level factors. Most of the factors identified were at the organizational/programme level. We did not identify any factor at the policy level.

#### Community level factors

We identified two motivators at the community level: deriving benefits from community work and making a difference. We did not identify any hygiene factor at the community level.

*Deriving benefits from community work*. Although supervisors are based at the offices of the CBOs, their work schedule require that they go to the community 4 days in a week to provide supervision to CHWs. Most of the supervisors explained that although they are based in the office, they visit the communities on a regular basis. One nurse supervisor expressed personal gratification that the community outreach component of their work afforded them the opportunity to develop rapport with patients and their families.

Am enjoying seeing patients because it’s nice to be in (in the office) and out (in the community) helping. We are helping now on one to one basis unlike in hospitals, where you have this patient and tomorrow they are going to be discharged and you don’t even know his/her name you just help and go. (Nurse/supervisor)

*Making a difference and community appreciation*. Participants felt motivated by the fact that their jobs provided them with the opportunity to make a difference in the lives of members of the community. They highlighted the various instances that their programme helped in improving the health and social condition of the community members that they serve. For supervisors, one of the measures of assessing the impact of their work on the community is the number of people whose lives are changed for the better.

The good part of this job is when you are counting people that you have helped; when you say I have helped that one and I have helped that one and people coming to you and appreciating. That’s the good part about it. (Manager/supervisor)

Supervisors also felt motivated by the show of appreciation from patients and other members of the community that they serve. They indicated that many of the community members showed appreciation by thanking them and acknowledging the value of their work in helping to improve their conditions.

To see the smile on the face of that child saying thank you for the plate of food that I gave her each and every day makes me proud to say I can feed the whole world by begging from different people. (Manager/supervisor)

#### Organizational level factors

We identified four motivators: promotion to supervisory position, acquisition of skills, taking opportunities to develop programmes and build capacity, and opportunity to advance career. We also identified hygiene factors: incentives that come with the position of supervisor.

*Promotion to supervisory position*. The promotion that many of the participants receive to become supervisors serves as a *motivator* for them. Majority of the supervisors who started out as volunteers felt ‘happy’, ‘blessed’ or ‘grateful’ for the opportunity to be promoted to the position of supervisor. Furthermore, the position comes with a number of opportunities which served as *hygiene factors*. Supervisors were gratified that the CBOs provided these incentives which include monetary incentives that they receive in this position which they did not receive in their previous position as CHWs. These incentives vary across CBOs depending on available resources with larger and better resourced CBOs providing more incentives. Monetary incentives include monthly salaries, yearly salary increments, retirement benefits, yearly performance bonuses, salary advances to cover educational expenses, financial support, educational loans and study leave.

*Acquisition of management skills and experience*. In some of the larger CBOs, participants receive induction training and learn new skills when they are employed. They also receive on-going training and acquire experience on the job which the participants saw as invaluable. As stated earlier, the skills training component of the job provides on-going experience. Training is provided each time supervisors are assigned new tasks.

So it helps to work in a community like this because I have faced challenges that a social worker or a nurse has not faced. So it really helps to start in a CBO because many things happen here… I have learnt a lot… a lot. (Supervisor)

Participants were also excited about the prospects of using the skills and experience gained to secure better paying jobs in the future.

I gain experience, so that if I get a (another) job they can pay me better. (Supervisor)

*Participation in the development of program*me*s and capacity building*. Supervisors provide training to CHWs within their own organization while a few of them had the opportunity to provide training to CHWs working in smaller CBOs, and they expressed gratitude for this opportunity. Many stated that they derived personal gratification from the opportunity to contribute to capacity building for CHWs.

The work is very rewarding and it’s something that I enjoy. I can finally say that I am in a job that I love to do. I love training people; I love empowering people and it is very gratifying. (Manager/supervisor)

They were also excited about the opportunity to participate in the development of new programmes to address community needs. As one participant put it: ‘so yah there is always something new that keeps you excited about what you are doing’.

*Educational advancement opportunities*. The CBOs also provided non-monetary incentives which served as motivators for participants. These include encouragement from line managers and board members to pursue their desires for educational advancement. Some of the organizations also gave supervisors study leave for personal development which enabled them to study for diplomas and degrees in tertiary institutions on a part-time basis. In one organization, for example, some of the supervisors received leave support to study for nursing diplomas, and degrees in social work and education.

### Factors contributing to demotivation and dissatisfaction

The factors identified as contributing to demotivation and dissatisfaction among supervisors are discussed under three overarching system level themes: community level factors, organizational level factors and policy level factors.

#### Community level factors

We identified only one *demotivator*: non-adherence to health advice, and one *dissatisfier*: working in communities with high crime prevalence.

*Non-adherence to health advice.* A majority of the participants felt frustrated and demotivated when they are not able to make a difference in their patients’ lives. Some of their patients defaulted in taking their antiretroviral medication while others failed to maintain healthy lifestyles as advised. One of the nurses expressed her frustrations.

It stresses you out if you don’t know what to do and whatever you are trying to do doesn’t work. It stresses you a lot. Like with HIV/AIDS although you can help as far as you can you don’t see any improvement. People default in treatment and though we try to help them not to default and to stick to the treatment, they don’t. [Adhere to treatment] (nurse/supervisor)

*Working in communities with high crime prevalence*. A real source of dissatisfaction for supervisors is their work context. CBOs work in communities with high levels of crime, and a few supervisors complained about the risk of sexual violence confronting both supervisors and CHWs. They also expressed the need to be vigilant while on visits to the community. A few of the supervisors indicated that the CHWs were generally afraid to work alone. Therefore, they encourage CHWs to go on home visits in pairs to provide support to each other. Supervisors working in CBOs that provide vehicles for supervisory visits identified the risk of losing their vehicles (and their lives) to robbers while driving in the communities where there is a high incidence of crime as a *dissatisfier*. A few of the supervisors indicated that they had experienced attempted robberies and/or witnessed other people being injured and/or robbed of their vehicles.

#### Organizational level factors

We identified five *dissatisfiers*: remuneration for CHWs and supervisors, problems with material and logistical resources, confronting job insecurity, dealing with work-related stressors, and working at the interface of CHWs and management. We also identified one *demotivator:* alienation from decision-making.

*Remuneration for CHWs and supervisors*. CHW stipends are sometimes irregular because of administrative challenges and reduction in funding allocation in the government funding agencies. The challenges with funding also meant that not all CHWs are paid stipends. Together, these contributed to attrition among CHWs and supervisors and also made it difficult for CBOs to attract and recruit new CHWs, which in turn led to shortage of supervisors and CHWs. Supervisors felt dissatisfied with having to deal with consequences of these challenges which undermines their work. A manager of a small CBO said:

Currently we have a staff shortage, only four of us here. Some people (CHWs) leave because we do not give stipends and people only work to get money at the end of the day. At the moment we need more hands so that we can try to help the community. (Manager/supervisor)

In addition, participants indicated that they were poorly remunerated when compared with colleagues working in government agencies. This caused some dissatisfaction with their jobs but did not seem to dampen their spirits or motivation and they continued to be motivated by other intrinsic factors.

Financially, the pay is not good but I always tell myself that whatever I am doing is helping in some way, somewhere, somehow. (Supervisor)

*Problems with material resources and transport*. All the participants reported that they were demoralized by the shortage of food items meant for needy households. In addition, only three of the CBOs had vehicles for delivering material supplies to their beneficiaries during supervisory visits. Therefore, supervisors had to deal with transport and logistical challenges.

We don’t have transport to help people; like in this organisation we need transport because we visit the patients and we use our cars. (Manager/supervisor)

*Confronting job insecurity*. Majority of the supervisors highlighted job insecurity as a source of dissatisfaction. The challenges with funding led to a situation where CBOs could not guarantee the job of supervisors and had to retrench some staff in order to stay afloat. All programme staffers were on 1-year contracts that are renewable subject to availability of funding. This means that when there are challenges with funding, CBOs can, and do, relieve supervisors of their jobs at the end of their contracts. These factors in turn led to job insecurity among supervisors with participants constantly living in fear of losing their jobs.

Some of our staff were retrenched which was quite sad because financially the organization could not afford to keep them.

*Work-related stressors*. The most common dissatisfier discussed by participants is work-related stressors. This mainly comes from work overload. Work overload is compounded by inadequate funding to hire supervisors and recruit CHWs. As a consequence, the few supervisors working in the CBOs had heavy workloads. Majority of the supervisors indicated that they sometimes felt overwhelmed with having to replace or fill in for absentee CHWs sometimes at short notice, conduct regular supervisory field visits and at the same time do a lot of administrative work. They found it daunting to combine the field visits, which they do 4 days a week, with administrative work. Additionally, managers in small CBOs reported work overload stemming from having to combine their managerial responsibilities which includes attending meetings, fundraising and overall management with the supervision of CHWs.

Sometime I have to go to Cape Town …for the whole weekend and then (the) next weekend I have to go somewhere else for a meeting. I don’t have time for my five children. I go home at 4.00 pm or 5.00 pm and I’m sleeping at 2.00 am trying to do the work that is supposed to be done. (Manager/supervisor)

*Navigating the interface between CHWs and management*. About half of the supervisors felt demotivated by having to bear the brunt of the frustrations of the CHWs about the shortage of materials, delay in the payment of stipends, lack of remuneration and lack of advancement opportunities. At the same time, supervisors have to account for the behaviour of CHWs who sometimes undermine their work.

Some CHWs let you down, stealing or not going to work. They tell you that they are going to work but they just go to those one room houses and stay there for the whole day and they come to you and they lie to you that they visited so many houses. It’s very sad. (Supervisor)

*Alienation from decision-making*. A major demotivating factor for some of the supervisors in three of the larger CBOs is the perceived alienation by the top management (board and manager) in decision-making. Supervisors claimed that the management make some important decisions without consulting them despite their wealth of experience. Participants argued that supervisors are better placed to provide invaluable contribution because of their first-hand experiences working in the community.

They (management) must be able to sit down with us as our superiors and involve us in decision making processes; get our input on situations because we understand the community better than they do because we work with the community on a daily basis. (Supervisor)

According to the participants, a consequence of alienation is that the management sometimes make impracticable decisions. Not only does the implementation of these decisions cause tensions between supervisors and management who resist these decisions, it also affects their relationship with CHWs and the community. One supervisor said with a sigh:

Uhmm! The challenge is with the management higher than my level. Sometimes they make some strategic decisions which are very stressful in terms of the decisions being made at that level and the implementation happening at my level. So if you don’t agree with the strategies it can be very challenging. (Supervisor)

#### Policy level factors

We identified two *hygiene factors* at the policy level: shortage of resources and funding for stipends and salaries and challenges of partnering with government agencies but did not identify any motivating factor.

*Shortage of resources and funding for stipends and salaries*. The major problem identified at the policy level is the failure of government agencies to provide sufficient funds for the operations of CBOs. This impacted on every aspect of CBOs’ operations at the organizational level. Participants complained that the shortage and irregularity of funding makes it difficult for the CBOs to meet their obligations of paying supervisor salaries and volunteer stipends on a regular basis. This also affects the ability of the organizations to provide services to their clients.

The management [CBO] is always complaining that the funding that they receive from government is not regular. Sometimes the government just cuts their funding without notice. (Supervisor)

*Challenges of partnering with government agency*. Participants discussed the challenges they encountered in working with the Sector Education and Training Authority (SETA): the government agency responsible for maintaining standards in education. They complained that bureaucratic delays make it difficult for them to secure approval for the curriculums that they developed for innovative training programmes in response to the needs in the community. In addition, the lack of government approval for in-house training curriculum that is not from government also makes it difficult for trainees to be accredited by government agencies. One supervisor who works as a training coordinator said:

I work with SETA and SETA is not the most supportive of institutions. They really delay the process and it’s that bureaucracy and time frames that almost hampered the program at some point. (Supervisor)

## Discussion

We drew on Herzberg’s motivation-hygiene theory to illuminate specific factors that could be critical for sustaining motivation and job satisfaction among supervisors. By doing this, we hoped to facilitate a deeper understanding of intrinsic factors that influence motivation (*motivators*) and the extrinsic factors that influence dissatisfaction (*hygiene factors*) among supervisors.

Our study shows that supervisors found a number of intrinsic *motivators* in their work which find expression in the terms that they used to describe their work: ‘meaningful’, ‘personally rewarding’ and ‘personally gratifying’. Intrinsic *motivators* include promotion from the position of CHW to that of supervisor, skills training and on-the-job experience, and the opportunity for personal development.

The finding that supervisors derive intrinsic motivation from being promoted to the position of supervisor provides support for the finding of previous studies among CHWs that show that the hope of acquiring skills and the need to pursue career paths within the health system are key motivations for CHWs in South Africa [10, 11, 17]. Most of the supervisors had only high school education when they enrolled as CHWs. Their work with CBOs provides opportunities for them to acquire skills and experience on the job. This together with the fact that CHWs come from marginalized communities with few opportunities for employment and income explains why acquisition of skills and the pursuit of career advancement are strong motivating factors for participants [10, 11, 17].

Yet, while supervisor positions in CBOs represent good opportunities for CHWs to learn new skills, advance their careers and earn an income, CBOs confront major funding problems that make it difficult to remunerate CHWs and support supervisor positions [7, 11]. A study among supervisors of community peer counsellors in South Africa showed that supervisors had to deal with attrition among community peer counsellors which the supervisors attributed to the low remuneration received by the CHWs [32]. Our study shows that funding problems undermine the ability of CBOs to pay for supervisor salaries and CHWs’ stipends and this leads to attrition among CHWs. CBOs in this study retrenched supervisors as a result of the shortage of funds with negative consequences for the work load and emotional wellbeing of the CHWs and supervisors who remain in the CBOs. These factors, together, constitute a source of dissatisfaction for supervisors in this study.

Globally, studies among CHWs identify attrition as a major factor undermining the sustainability of CHW programmes [2, 17–19]. Recent studies on CHWs in South Africa have highlighted the link between CHW motivations and attrition, and a number of studies have sought to gain deeper and more nuanced understanding of CHW motivations and factors influencing sustained volunteer CHW motivations in order to better inform policies intended to address the problem of CHW attrition [11, 17]. Dageid and colleagues argue that an important motivation for sustained volunteering among CHWs is the hope to secure a more stable position in the health and social services sectors and that this motivation develops over time as CHWs acquire skills and experience which leads to a desire for professional acknowledgement, job benefits and paid employment.

The findings of our study on supervisors support the findings of previous studies exploring the motivation of CHWs [2, 11, 15, 17, 19]. It demonstrates that many of our study participants were promoted from CHWs to supervisor positions, and this fulfils their motivations to secure paid jobs. However, the precarious funding situation confronting CBOs leads to job insecurity for supervisors, a factor that causes dissatisfaction. This, among other factors, makes it difficult for supervisors to ‘rest on their oars’. It therefore makes sense that supervisors continue to seek for paths to jobs that are more regular and more secure in the health and social services sectors [9, 11]. Our finding shows that the quest for career advancement among CHWs does not stop with promotion to paid supervisor positions in their organizations but supervisors also continue to be motivated to acquire higher-level skills to become nurses, social workers, community developers or some other high-skilled professional in the health and social services sectors. As our study demonstrates, the position that supervisors occupy provides them with the platform to, in addition to acquiring skills and experience, pursue opportunities for further training and professional qualification that improve their skills and employability in more stable positions in the health sector.

Some authors have argued that the context of poverty and unemployment in which many community care worker programmes are situated influences CHW motivations [11, 17]. We argue that it is reasonable for supervisors who have limited skills, come from scarce resource settings and are acutely aware of the precarious funding situation confronting CBOs to want to pursue a career path which offers job stability and security in the health and social services sectors. The desire for secured positions among supervisors who participated in this study finds expression in their pursuit of further education in nursing, social work and education fields.

Following Herzberg’s theory, CHW programmes should offer opportunities for training and promotion of CHWs to the position of supervisors; this could provide a platform for empowering women from marginalized communities—who constitute the majority of CHWs offering unpaid labour—with skilled jobs [11, 17] over the medium to long term. A failure to provide opportunities for CHWs to satisfy this motivation will not only lead to demotivation and low performance and productivity but could also lead to attrition among CHWs [22, 23]. Our findings support growing calls for health policy-makers to create career paths for CHWs [9, 11, 17, 33]. In addition, we argue that primary health care policy should not only be limited to the creation of career paths that enable CHWs to be promoted to the position of CHW supervisor but should also provide an opportunity for supervisors to move up to mid-level health care cadres such as nurses and auxiliary social workers [34].

Supervisors in previous studies reported insufficient skills training to handle supervisory tasks [16, 21, 32]. Our study, on the other hand, showed that supervisors were motivated by the supervisory skills received. Participants did not mention any deficiency in training which one could interpret to mean that they were satisfied with the training provided to function in the position of supervisors. On the other hand, it could be because the focus of our questions was on factors influencing motivation and job satisfaction and not specifically on their experiences of skills training and practice. Research focusing on supervisors’ experiences relating to their training and practice will be an invaluable addition to the literature on supervisors in CHW programmes.

The opportunity to earn an income is a hygiene factor that helped to prevent dissatisfaction among supervisors. Nonetheless, supervisors were dissatisfied with their low income but appeared determined to not allow it to demotivate them. Instead, supervisors kept their focus on intrinsic factors - making a difference, opportunities for development of managerial skills, building capacity of CHWs and career advancement opportunities - that enabled them to stay motivated. Thus, as discussed earlier, the opportunity for career advancement and hopes of a better job were strong motivations for not quitting despite their low remuneration. This finding supports Herzberg’s theory, which argues that the absence of hygiene factors (presence of *dissatisfiers*) does not lead to demotivation but to dissatisfaction [22] and suggests the need for policy-makers to not only address the remuneration of supervisors but to also provide opportunities for them to satisfy their motivations for educational and career advancement.

Previous studies have identified issues with supplies and transport as major problems confronting CHW programmes globally [9, 10, 15, 32, 35]. Supervisors in our study identified challenges with availability of material supplies as well as transportation and related logistical problems as major sources of dissatisfaction. Our findings have important implications for existing CBO-run CHW programmes as well as countries intending to develop national CHW programmes. Both these models require technical and financial resources in order to be sustainable.

A study among supervisors in Mozambique found that supervisors found it difficult to solve issues relating to material supplies and remuneration because of a lack of administrative power [16]. Our finding shows that supervisors find it challenging working at the nexus of CHWs and management. They felt demotivated by having to deal with the shortage of materials and unfavourable work conditions of the CHWs and to advocate for CHWs at the management level. At the same time, supervisors had to oversee the implementation of CBOs’ policies and manage CHWs who may sometimes undermine their work. On the one hand, CHWs implement management decisions at the grassroots level by supervising CHWs who provide health and social services in the communities. On the other hand, they help solve problems confronting CHWs and convey CHWs’ concerns as well as that of patients and service users to management. Therefore, supervisors occupy a unique and critical position within CBOs as they are able to provide first-hand assessment of the delivery of health care services to management and at the same time gain unique insights into CHWs experiences, challenges and concerns [16]. Supervisors are therefore an indispensable bridge between policy-makers, programme planners and management on the one hand and the CHWs and communities on the other.

Given these considerations and the link between supervision, retention, performance and productivity of CHWs, and sustainability of CHW programmes [16, 20, 21, 35], we call on policy-makers and researchers to pay particular attention to understanding perspectives of supervisors in order to gain better insights into factors related to CHWs, supervisors and CBO management that could inform the development and sustainability of national CHW programmes. In addition, CHW programmes could benefit immensely by drawing on supervisors’ insights and offering them greater managerial responsibilities that allow them to participate in programme development and contribute to decision-making at the management level. These measures could in turn help improve the quality of decision-making, work relations, and the development of managerial skills, experience and competencies among supervisors. All these interventions could have positive knock-on effects on supervisor motivations and insights from supervisors could help inform policy and programmes aimed at improving retention of CHWs as well as the sustainability of CHW programmes [20].

Our findings have implications for the global drive to scale-up CHW programmes and to strengthen health systems. Given the potential value of supervisors, it seems reasonable for national CHW programmes to budget for the funding of supervisor positions. Yet the scaling-up of CHW programmes in some African countries has been fraught with funding problems. In Kenya, for example, a policy decision to remunerate CHWs had to be scaled back because of insufficient funding [36]. And in South Africa, government’s efforts to remunerate CHWs and create career paths executed through a public works programme had mixed success [34, 37], (Parenzee and Budlender: South Africa’s expanded public works programme: exploratory research for the social sector, unpublished). While the programme provided funding to CBOs to remunerate a large number of CHWs, an even larger number of CHWs did not have the opportunity to participate in the programme [33, 34], (Fridman, Behngu, Mothibe, Reynolds & Mafuleka: Executive Summary: scaling up the EPWP social cluster, unpublished). Moreover, the programme encountered challenges in building the capacity of CHW to facilitate skills acquisition to pursue career paths as mid-level skilled health care workers because of insufficient trainers [33, 34, 37], (Parenzee and Budlender: South Africa’s expanded public works programme: exploratory research for the social sector, unpublished).

The South African PHC re-engineering model provides for outreach teams operating from clinics at the ward level. Each team comprises CHWs, environmental health practitioners and health promoters who provide outreach services to communities under the supervision of a professional nurse. Supervisors working in CBOs could play a central role in this PHC initiative. Yet, the PHC re-engineering policy does not make explicit, the role of CBOs in the new PHC outreach model [6, 9, 12] much less that of supervisors affiliated to CBOs. It therefore seems expedient for policy-makers to work with other stakeholders to explore how to make use of the skills of supervisors who are affiliated to the numerous CBOs working in marginalized communities across the country in the new PHC initiative. This should involve making use of supervisors’ skills at the community (grassroots) level through innovative formalized collaborative models.

Further, researchers have expressed concern that the new PHC initiative might affect the funding of CBOs negatively. i.e. there is concern that government funding agencies as well as donor agencies who provide funding support to governments in order for them to support CBOs may channel their funds to the PHC re-engineering initiative thereby denying CBOs of much needed financial and technical support [9, 12]. These highlight the need for policy-makers and programme planners to explore how to make certain that the PHC re-engineering initiative benefits from the vast experience of supervisors in CBOs and to ensure that CBOs receive regular funding and technical support from relevant government agencies in order to address their funding problems and deliver critical health and social services to marginalized communities.

Our study suggests that it is worthwhile for policy-makers and stakeholders globally and particularly in LMICs to explore a broader conceptualization of the role of CHW programmes to encompass health systems strengthening as well as economic development of marginalized communities. We argue that CHW programmes should be conceptualized with the dual purpose of strengthening health systems for health services delivery and creating jobs for citizens in poor and marginalized communities who currently carry the burden of providing unpaid community health care particularly in LMICs. In this regard, policy-makers and programme planners need to explore the possibility of developing and budgeting for models of national CHW programmes that help build capacity to address health care worker shortages in health systems at the primary care level and create jobs in poor communities in LMICs. This could be achieved through creating paid positions for supervisors at the community level and providing high quality training to supervisors to help in the delivery of community health services. In addition, there is a need for national governments to support formal training of supervisors to progress to mid-level health care workers such as primary health care nurses [34]. In addition to strengthening health systems, this approach could help reduce unemployment and poverty in marginalized communities with potential multiplier ramifications for these communities and the economy at large [34].

The limitations of this study should be considered when interpreting the findings. First, the use of a snowball sampling technique may have led to an overrepresentation of CBOs in the same network that may have similar experiences. Thus the experiences of participants might not adequately reflect that of all supervisors working in CBOs. However, we addressed this potential problem by including a range of CBOs working in a range of community settings in order to include diverse perspectives from supervisors from diverse CBOs. Second, all the supervisors in our study were employed by CBOs who have limited resources. The experiences of supervisors employed by government in formal primary health care settings such as primary health care clinics might differ. A third limitation is the fact that we did not explore the perspectives of CHWs and managers of CBOs in order to gain more insight into the motivations of supervisors.

## Conclusions

Given that supervisors are in a unique position to identify issues that are critical to the success of CHW programmes, policy-makers must pay attention to factors that could sustain job satisfaction and motivation among supervisors in CHW programmes. This could help improve effectiveness and sustainability of CHW initiatives. There is also a need to build the capacity of supervisors to address health worker shortages in primary health care in South Africa and globally and particularly in LMICs aiming to formalize and scale-up the use of CHWs in their national health systems. Finally, it is critical for policy-makers and researchers to seek to explore the perspectives of supervisors in order to gain deeper and richer insights into CHW programmes which will help inform more appropriate policies and planning for CHW programmes globally.

## Abbreviations

CHW: Community health worker
DOH: Department of Health
LMIC: Low- and middle-income countries
PHC: Primary health care
